# Green-synthesized gold nanoparticles from black tea extract enhance the chemosensitivity of doxorubicin in HCT116 cells *via* a ROS-dependent pathway†

**DOI:** 10.1039/d1ra08374k

**Published:** 2022-03-23

**Authors:** Tanushree Das, Snehasis Mishra, Sayoni Nag, Krishna Das Saha

**Affiliations:** Cancer Biology & Inflammatory Disorder Division, CSIR-Indian Institute of Chemical Biology Kolkata-700032 West Bengal India Krishna@iicb.res.in +91-33-2473 5197 +91-33-2499 5810

## Abstract

Green gold nanoparticles (GNPs) were prepared from black tea extract (BTE) and used to examine the chemosensitivity of doxorubicin in colon cancer cell line HCT116. BTE-GNPs were prepared by a single-step method and characterized by UV-Vis spectroscopy, FTIR spectroscopy, SEM, DLS and zeta-potential. The MTT assay was performed to determine the cytotoxicity of HCT116 cells and also normal kidney cells HEK293. Apoptosis and ROS generation were investigated by flow cytometry. The inhibition of ROS levels by the inhibitor NAC was determined by both spectrofluorimetry and confocal microscopy. Expression levels of pro- and anti-apoptotic proteins were determined by a western blot technique. BTE-GNPs significantly enhanced the cytotoxic effect of DOX with its co-treatment in HCT116 cells. The cytotoxic effect of BTE-GNP + DOX was involved in apoptosis *via* a ROS-dependent pathway by enhancing the pro-apoptotic protein expression. Therefore, our results indicated that green gold nanoparticles of black tea extract (BTE-GNP) may be potent chemosensitizers of doxorubicin.

## Abstract 2

Green gold nanoparticles (GNPs) were prepared from black tea extract (BTE) and used to examine the chemosensitivity of doxorubicin in colon cancer cell line HCT116.

## Graphical abstract

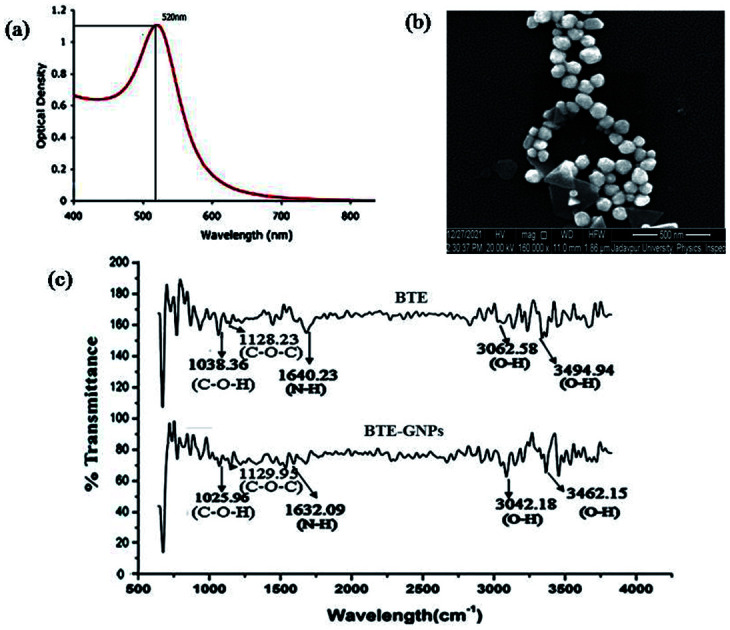

## 1. Introduction

Colon cancer is the third most common malignancy of the gastrointestinal tract among men and women, and this leads to 50% of cancer-associated mortality world-wide.^1^ It was in 2012, when about 1.4 million individuals were newly diagnosed with CRC, and the mortality rate was about 693 900.^2^ Chemosensitization means the use of a drug to enhance the activity of another by modulating one or more mechanisms of anticancer activity. Such combination of more than one drug exhibits higher therapeutic efficacy at lower doses than mono-therapy. The combination of drugs acts synergistically or in an additive manner.^3^ In addition to radiation and surgery, chemotherapy is important in curing cancer. However, chemotherapy also has several side effects upon its high doses.^4^ Thrombocytopenia, neutropenia, and anemia are the side effects of chemotherapy.^5^ Combined treatment of more than one anticancer agents that target different pathways or act in an additive manner may solve the limitations of high doses of monotherapy.^6^ One strategy is combination of chemotherapy agents with natural anticancer agents such as polyphenols.^7^ Black tea (*Camellia sinensis*) is one of the most widely consumed beverages that contained high levels of polyphenols.^8^ The anti-cancer activity of black tea-containing polyphenols is well documented in both *in vitro* and preclinical studies.^9^ Gold nanoparticles have been proved to be efficient anticancer agents.^10^ Due to its biocompatibility and noncytotoxicity, it is now being widely used for combinatorial cancer therapy strategies.^11^ In the present study, we aimed to synthesize green gold nano particles using black tea and examine its combinatorial effect on doxorubicin in a human colon cancer cell line, HCT116.

Nanoparticles such as gold nanoparticles usually act in the range of 1–100 nm. In recent years, gold nanoparticles have been included in areas such as advanced nanomedicine, drug development and delivery. Water-soluble gold nanoparticles (AuNPs) act upon several diseases including cancer, hepatitis, and neurodegenerative diseases. AuNPs are beneficial due to certain features such as biocompatibility, lower toxicity, ability to penetrate cells, specific bio-distribution, specific targeting, aqueous solubility and good therapeutic indices.^12^ Now a days it has been a high concern to develop non-toxic synthesis methods, and therefore, green chemistry becomes highlighted. The biological green synthesis of gold nano particles became the focus of research nowadays. Green synthesized AuNPs were eco-friendly and cost-effective than other chemical and physical methods using hazardous reagents and solvents as reducing agents, which ultimately caused to improve the efficiency and enhanced the design of non-toxic products.^13^ Such alternative biosynthetic green methods that utilized the plant-based phytochemicals could reduce the metal ions referred to as the “green nanotechnology”.^14^ Green synthesis has overcome the different limitations over chemical and physical methods due to various factors, as it is cost-effective, environment-friendly, and easily available. By that way, nanoparticle synthesis becomes cheaper, does not require any high pressure, energy, and temperature and have no toxicity.^15^

Many studies have revealed that ROS is the tumor suppressive agent.^16^ Various chemotherapeutics enhance the ROS production that triggers the cell death.^17^ ROS induces programmed cell death (apoptosis) in cancer cells.^18^ Several *in vitro* studies have been conducted, after incubation of DOX became activated in cells.^19^ ROS is one of the factors of cancer cell death mediated by doxorubicin (DOX).^20^ A gold nanoparticle induces cancer cell death through higher levels of ROS generation.^21^ Generally, the apoptotic signal of ROS causes the activation of caspase-9 (effector caspase) and caspase-3 (executioner caspase) by engaging BAX to the mitochondria, releasing cytochrome-*c* to cytosol, and promoting cleavage of PARP.^22^ Herein, we show that BTE-GNP combined with DOX enhanced the cell-cytotoxicity *via* elevated ROS generation. Our study also suggests that BTE-GNP improves the cytotoxicity of DOX at its low doses.

## 2. Materials and methods

### 2.1 Materials

3-(4,5-Dimethylthiazol-2-yl)-2,5-diphenyltetrazolium bromide (MTT) is the product of SRL, *N*-acetyl cysteine (NAC) was purchased from Sigma Aldrich, and HCT116 and HEK293 cell lines were obtained from the National Centre For Cell Science (NCCS), Pune, Govt. of India. HCT 116 cell lines were cultured in DMEM Dulbecco's Modified Eagle's Medium (DMEM), supplemented with 10% fetal bovine serum (FBS) and 1% penicillin and streptomycin antibiotics, where trypsin and ethylenediaminetetraacetic acid (EDTA) were all purchased from Gibco BRL. Tissue culture plastic wares were obtained from NUNC (Roskidle, Denmark). DAPI (4′,6-diamidino-2-phenylindole dihydrochloride), acridine orange (AO), and ethidium bromide (EtBr) were obtained from Invitrogen (California) and BCA protein assay reagent from Fermentus (EU). The primary antibodies for Bcl2, Bax, caspase 3, cleaved caspase-3, caspase-9, cleaved caspase-9, cytochrome *c*, P53, cleaved PARP, and β-actin were obtained from Santa Cruz Biotechnology (Santa Cruz, CA). DCF-DA was obtained from Sigma-Aldrich (MO, USA). An Annexin-V FITC apoptosis detection kit was obtained from Calbiochem (CA, USA). Doxorubicin was purchased from Sigma Aldrich.

### 2.2 Cell line and culture condition

Both the HCT116 and HEK293 cell lines were cultured in DMEM supplemented with 10% heat-inactivated FBS at 37 °C with 5% CO_2_ in a humidified atmosphere, where 100 units per ml penicillin/streptomycin was also used. Cells were seeded in T25 flasks at appropriate densities and about 80–90% confluent cells were used for the experiments. Later when the cells were over confluent, they were transferred to the T75 flasks.

### 2.3 Synthesis of AuNPS

A stock solution of 10 mM of AuNPs was prepared by a simple method. Briefly first 150 mg of black tea leaf was boiled at 20 °C with 15 ml of distilled water (DI) for 30 min and then filtered with a Millipore filter paper (0.2 μM). In another flask, a suitable amount of HAucl_4_, 3H_2_O was dissolved in 15 ml of water for further 30 min at 100 °C under magnetic stirring to produce 1 mM of dilute working solution of gold. To carry out the reduction process from Au^3+^ to Au^0^ reaction, the black tea solution was poured drop-wise into 15 ml of 1 mM working dilute gold solution, and the reaction mixture was stirred for 5 min to get the reduced pale pink colored Au^0^ solution. The reaction mixture was stirred for another 10 min. Thus, the gold nanoparticles were formed and then filtered with a 0.2 micron filter.

### 2.4 Characterization of NPs

#### 2.4.1 UV-visible spectra analysis

The characteristic absorbance band of AuNPs shows that it depends entirely upon the size of the nanoparticles. UV-Visible spectra from 400 nm to 700 nm of AuNPs were recorded using a UV-Vis spectrophotometer (Lambda 35, PerkinElmer).

#### 2.4.2 Scanning electron microscopic (SEM) analysis

The size and the surface morphology of the BTE-GNPs were analyzed using an FE-SEM technique. Briefly, the as-synthesized BTE-GNPs were diluted and dispersed by ultrasonication for about 15 min, air-dried and then mounted on silica specimen stubs. The images were captured using a JEOL JEM 6700 field emission scanning electron microscope (FE-SEM) by applying an electron accelerated voltage of 20 kV.

### 2.5 Fourier transform infrared (FT-IR) spectral studies

The possible chemical functional groups of both the black tea extract and the BTE-GNPs were analyzed using a PerkinElmer FT-IR spectrometer (MA, USA). For the analysis, the samples were shacked in a shaker for 30 s. The FTIR spectrum was measured in the range between 400 and 4000^−1^.

### 2.6 Analysis of hydrodynamic diameter (nm), zetapotential, and polydispersity index (PDI) at different pH levels

The mean nanoparticle diameter (hydrodynamic size) and polydispersity index were analyzed by differential light scattering (DLS), which were obtained using 12 mm cells at 90° angle and 25 °C. A Zetasizer 3000 HSA (Malvern Instruments, Malvern, UK) was used to analyse the zeta potential. Nanoparticles (BTE-GNPs) were suspended at different pH levels as acidic (pH 5.5), PBS (pH 7.4) and basic (pH 9.5) buffer solutions before the measurements and 400 μl was loaded into the cuvette for DLS.

### 2.7 Cell cytotoxicity assay

The viability of HCT116 cells was measured by 3-(4,5-dimethylthiazol-2-yl)-2,5-diphenyltetrazolium bromide (MTT) assay. In brief, the HCT116 cells and also normal cells HEK293 were plated in 96-well plates in triplicate manner and treated with different concentrations of BTE-GNP and DOX individually or in their combined form for 24 h. In this experiment, the time-dependent assay had also been done. After treatment, MTT (40 μl from 5 mg ml^−1^ stock solution in PBS) was added and incubated at 37 °C for 4 hours. The purple colored formazan crystal was dissolved into 80 μl of DMSO. The optical density was measured at 595 nm using an ELISA (enzyme-linked immune sorbent assay) reader. The inhibition of cell growth of treated cells was compared to the control or untreated cells. The IC_50_ value was also determined. In this study, cells were pretreated with NAC (1 mM) and then treated with both BTE-GNP and DOX alone and in combination, and then the cell viability was quantified.

### 2.8 Assessment of cellular death parameters under a microscope

Cells (1 × 10^6^ per well) were seeded and grown in 35 mm culture plates, and after that, 80–90% confluency was reached upon treatment with BTE-GNP and DOX in combined form with their respective IC_20_ doses for 24 h. To observe nuclear damage or chromatin condensation, cells were stained with 10 μg ml^−1^ of DAPI, incubated for 30 min and viewed under a fluorescent microscope. Cells were also stained with acridine orange (AO) and ethidium bromide (EtBr) to differentiate between apoptotic/necrotic death. Cells were imagined under a fluorescent microscope (OLYMPUS IX70, Olympus Optical Co. Ltd, Tokyo, Japan) and images were acquired with excitation with 488 nm and emission with 550 nm wavelengths.

### 2.9 Quantification of apoptosis by flow cytometry

Apoptosis of colon carcinoma HCT116 cells was determined using an Annexin-V FITC apoptosis detection kit (Calbiochem, CA, USA). At first, the cells were treated with BTE-GNP and DOX alone as well as in combined doses, and then cells were stained with FITC-coupled Annexin V-PI as per manufacturer^'^s instruction. The live, early and late apoptotic and necrotic percentages of cells were determined by flow cytometry (Beckton Dickinson, San Jose, CA, USA). In this study, triplicate assay were performed.

### 2.10 Intracellular ROS generation assay

ROS generation was examined by DCF-DA. In the presence or absence of 0.5 mM and 1 mM of NAC, cells were treated with BTE-GNP and DOX alone or in combined form for 24 h, and then cells were washed with PBS and incubated with 10 mM DCF-DA for 15 min at 37 °C. Intracellular ROS that mediated the oxidation of DCFH is cleaved to produce the fluorescent 2′,7′-dichlorofluorescein (DCF) in HCT116 cells, which was observed using a spectrofluorimeter, flow cytometer (Becton Dickinson, San Jose, CA, USA) and also a confocal microscope with excitation and emission wavelengths of 480 nm at 525 nm respectively.

### 2.11 western blot analysis

The different protein expression levels were determined by western blot analysis. In brief, either treated or untreated cells and also both adherent and floating cells were collected and lysed with a lysis buffer for 30 min on ice. The supernatant protein (30 μg of protein) was loaded and separated in 10% sodium dodecyl sulphate polyacrylamide gel electrophoresis (SDS-PAGE) and transferred onto a PVDF membrane. Furthermore, membranes were blocked with 5% BSA and then incubated with primary antibodies (1 : 3000) for BAX, Bcl2, PARP, cleaved PARP, cyto-*c*, cleaved caspase-3, cleaved caspase-9, p53, P-p53, and β-actin overnight at 4 °C. Blots were then washed three times with TBST containing 0.1% (w/v) tween 20, pH 7.6, and after that incubated with horseradish peroxidase-conjugated secondary antibodies (1 : 5000) in a shaker at room temperature for 2 h. After that, the membranes were washed for 4 times and then finally protein expressions were visualized using the horseradish peroxidase substrate (peroxides/luminal) in a chemidoc system. The Image-j software was used to analyze the quantification of proteins.

### 2.12 Statistical analysis

All values are expressed here as mean ± sd. Using the GraphPad Prism software (CA, USA), statistical significance was calculated between control and treatment groups, and these were analyzed using a one-way analysis of variance (ANOVA). *P* < 0.05 was considered to be statistically significant.

## 3. Results

### 3.1 Synthesis and characterization of black tea extract reduced gold nanoparticles (GNPs)

**Fig. 1:**
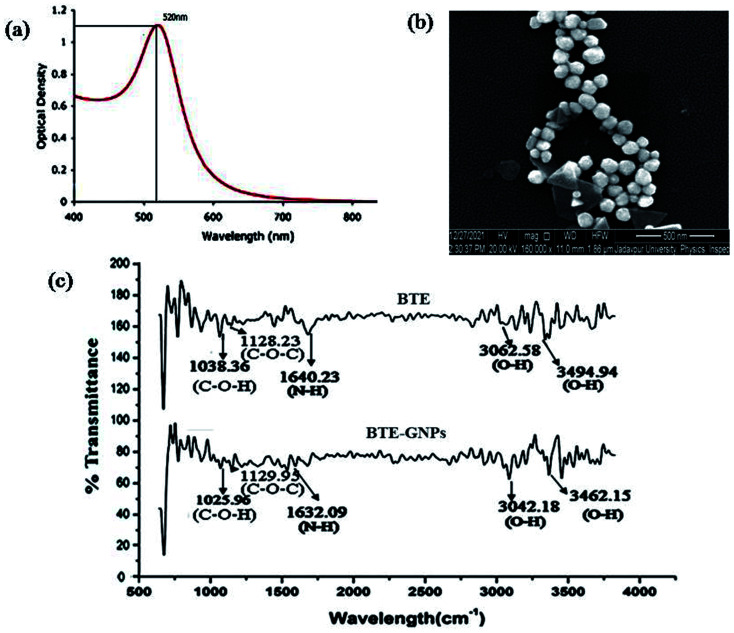
Characterization of BTE-GNP nanoparticles. (a) UV-visible absorption spectra for the reduced gold nanoparticles (GNPs) of black tea extract (BTE), (b) represents the SEM image of the BTE-GNP. (c) FT-IR spectra of both black tea extract (BTE) and black tea extract reduced gold nanoparticles (BTE-GNPs).

The present result indicated the green synthesis of gold nano particles (GNPs) using the black tea extract (BTE). An aqueous solution of hydrogen tetrachloroaurate (HAuCl_4_) was reduced by the black tea leaf extract (BTE) to formulate the GNPs. The formation of GNPs was detected by color changes in the reactive solution mixture, where the gold solution was reduced with a plant black tea leaf extract. HAucl_4_ in an aqueous solution remained yellow colored, which means that Au^3+^ was not reduced. When 1 ml of black tea extract was added, its color changed from pale yellow to pale pink. This indicated the reduction of Au^3+^ to Au^0^. This observation illustrated the reduction and capping or stabilization of newly formed metal gold nanoparticles (Au NPs) using the tea leaf extract. Gold nanoparticles are known to have at maximum absorbance in the range of *λ*_max_ 400 to 700 nm. We measured the absorbance of BTE-reduced GNP (BTE-GNP) using a UV-visible spectrophotometer (Lambda 35, PerkinElmer) and BTE-GNP showed a characteristic absorbance peak at 529 nm, indicating the formation of gold nanoparticles (Fig. 1a). We have further confirmed the shape of formulated reduced gold nanoparticles by Scanning Electron Microscopy (SEM) (Fig. 1b). The SEM gives a surface morphology of the particles; and therefore, it was possible to know that the particles were circular and spherical in shape.

The FTIR spectra of BTE-GNP and BTE showed the possible attachment of the functional groups, as shown in Fig. 1c. The FTIR spectrum of the black tea extract (BTE) expressed the peaks at 3494.94 cm^−1^ (O–H stretching of carboxylic acid, strong peak intensity), 3062.58 cm^−1^ (O–H stretching of carboxylic acid, a medium peak intensity), 1640.23 cm^−1^ (N–H bend of amines, strong peak intensity), 1128.23 cm^−1^ (C–O–C stretching of polysaccharides, strong peak intensity), and 1038.36 cm^−1^ (C–O–H stretching of amino acids, strong peak intensity). The FTIR spectrum of the gold nanoparticles (Fig. 1c) indicated the peaks at 3462.15 cm^−1^ (O–H stretching of carboxylic acid, strong peak intensity), 3042.15 cm^−1^ (O–H stretch of carboxylic acids, with strong peak intensity), 1632.09 cm^−1^ (N–H stretching of amines, medium peak intensity), 1129.95 cm^−1^ (C–O–C stretching of polysaccharides, strong peak intensity), and 1025.96 cm^−1^ (C–O–H stretching of amino acids, strong peak intensity). Here, it can be postulated that functional groups such as O–H, N–H, C–O–C, and COOH may be responsible for the reduction of HAucl_4_ to convert into reduced gold nano particles. On the basis of comparison of 2942.18 cm^−1^, 2942.18 cm^−1^, and 2859.64 cm^−1^, these peaks of BTE-GNP slightly shift from the peaks of black tea extract suggest the presence of the same functional groups in BTE and BTE-GNPs.

In a time-dependent manner, the hydrodynamic size (nm) and also zeta potentials of the BTE-GNPs were analyzed (Table 1). The hydrodynamic size of the BTE-GNP under pH range 5.5 (acidic buffer) was 56.1 ± 1.1 nm at 12 h, 76.3 ± 1.8 nm at 24 h, and 98.6 ± 2.1 nm at 36 h. Where the hydrodynamic size under the pH range from 7.4 (PBS) to pH 9.5 (basic buffer) were 33.4 ± 0.8 at 12 h, 55.6 ± 1.2 nm at 24 h, 75.6 ± 1.5 at 36 h, and then increased as 66.2 ± 1.6 nm at 12 h, 92.5 ± 2.0 nm at 24 h and 112.5 ± 2.5 nm at 36 h respectively. The zeta potential was used to measure the surface charges of the BTE-GNP under various pH ranges that could act as a marker for NP stability. The result indicated the zeta potentials of the acidic buffer contained BTE-GNP as −12.5 ± 0.1 at 12 h, −20.9 ± 0.7 at 24 h and −25.2 ± 1.1 at 36 h. Under PBS buffer suspension showed −6.56 ± 0.01 at 12 h, −9.45 ± 0.09 at 24 h and −13.5 ± 0.3 at 36 h, whereas under basic buffer suspensions showed −15.3 ± 0.4 at 12 h, −18.7 ± 0.6 at 24 h and −29.6 ± 1.5 at 36 h, which indicated the stable form of BTE-GNPs at different pH levels. The polydispersity index (PDI) gives the information about the uniform size distribution of NPs. The decreased value of PDI was considered to be monodisperse and uniform in size.^23^ Here, the estimated PDI result (Table 1) of the BTE-GNP showed 0.29 at 12 h, 0.33 at 24 h, and 0.36 at 36 h under an acidic buffer solution, where under PBS solution showed 0.31 at 12 h, 0.34 at 24 h, and 0.38 at 36 h. PDI under basic buffer solutions were 0.35 at 12 h, 0.39 at 24 h and 0.44 at 36 h, which suggested some polydispersity under acidic and PBS buffers but a minor increase in the basic buffer.

**Table 1:** Some physical characters of BTE-GNPs^a^

AuNPs (BTE-GNPs) in different pH	Hydro-dynamic diameter (nm) on 12 h	Hydro-dynamic diameter (nm) on 24 h	Hydro-dynamic diameter (nm) on 36 h	Zeta-potential (mV) on 12 h	Zeta-potential (mV) on 24 h	Zeta-potential (mV) on 36 h	Poly-dispersity index (PDI) on 12 h	Poly-dispersity index (PDI) on 24 h	Poly-dispersity index (PDI) on 36 h
BTE-GNPs in acidic buffer (pH 5.5)	56.1 ± 1.1	76.3 ± 1.8	98.6 ± 2.1	−12.5 ± 0.1	−20.9 ± 0.7	−25.2 ± 1.1	0.290	0.330	0.360
BTE-GNPs in PBS (pH 7.4)	33.4 ± 0.8	55.6 ± 1.2	75.6 ± 1.5	−6.56 ± 0.01	−9.45 ± 0.09	−13.5 ± 0.3	0.310	0.340	0.380
BTE-GNPs in basic buffer (pH 9.5)	66.2 ± 1.6	92.5 ± 2.0	112.5 ± 2.5	−15.3 ± 0.4	−18.7 ± 0.6	−29.6 ± 1.5	0.350	0.390	0.440

a Represents the time dependent values of hydrodynamic diameter (nm), zeta-potential, and polydispersity index (PDI) of BTE-GNPs (AuNPs) suspended in acidic buffer, in PBS, and in basic buffer respectively.

### 3.2 Effect of BTE-GNP and DOX on cell-proliferation assay on colon cancer cells, HCT116, and also on normal kidney HEK293 cells

**Fig. 2:**
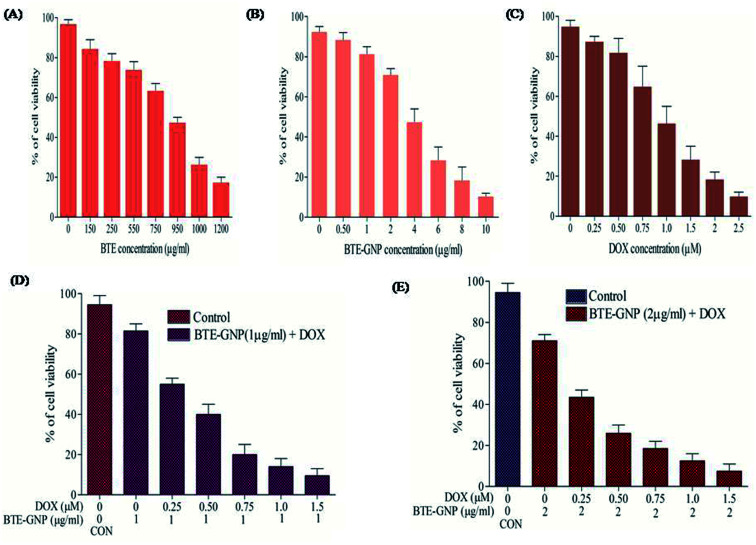
BTE, BTE-GNP, DOX, BTE-GNP + DOX decreased cell viability of HCT116 cells. HCT116 cells were treated with different concentrations (a) (0–1200 μg ml^−1^) of free black tea extract only, (b) (0–10 μg ml^−1^) of black tea extract-gold nano particle (BTE-GNP), and (c) doxorubicin (DOX) with (0–2.5 μm), (d) BTE-GNP (1 μg ml^−1^) + indicated doses of DOX for 24 h. (e) BTE-GNP (2 μg ml^−1^) + indicated doses of DOX for 24 h. Cytotoxicity were determined by MTT assay. Here data represented are the mean ± standard deviation of three independent experiments.

**Fig. 3:**
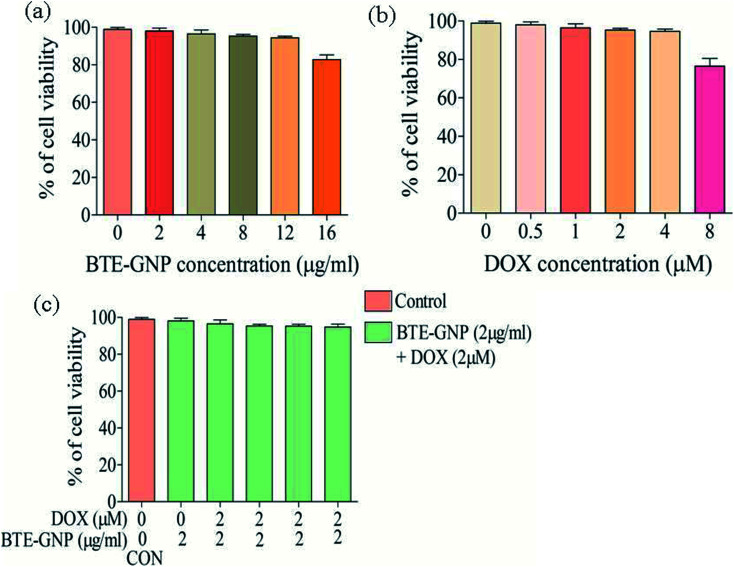
Normal cell line HEK293 were treated with BTE-GNP, DOX, BTE-GNP + DOX shown increased cell survival, lower cell cytotoxicity. HEK293 cells were treated with different concentrations (a) (0–16 μg ml^−1^) of black tea extract-gold nano particle (BTE-GNP), (b) doxorubicin (DOX) with (0–8 μm), (c) BTE-GNP (2 μg ml^−1^) + indicated doses of DOX for 24 h. Cytotoxicity were determined by MTT assay. Here data represented are the mean ± standard deviation of three independent experiments.

In the present study, cytotoxic effects of BTE, BTE-GNP and DOX on colorectal cancer cell line, HCT116, were studied by the MTT assay. Cell growth in 24 h was inhibited by all these three compounds in a dose-dependent manner (Fig. 2A–C). Growth inhibition by BTE-GNPs was nearly 200 times higher than BTE. Treatment of BTE-GNPs with DOX remarkably increased its cytotoxicity (Fig. 2D). Treatment of IC_20_ concentration of BTE-GNP (1 μg ml^−1^) with IC_20_ of DOX resulted in about 60% cytotoxicity. There was about 85% cytotoxicity of the combination of IC_20_ of BTE-GNP with IC_30_ of DOX (0.75 μM). Similarly, enhancement in cytotoxicity was observed following treatment with IC_30_ of BTE-GNP (2 μg ml^−1^) and DOX (Fig. 2E). Thus, the data confirms that BTE-GNP can enhance the cytotoxic effect of DOX. Both the single and combined cytotoxic effects of DOX and BTE-GNP on normal cell line HEK293 were also determined by the MTT assay. Cells were grown for 24 h and treated in a dose-dependent manner (Fig. 3). The treatment of the highest dose of BTE-GNPs (16 μg ml^−1^) caused cytotoxicity of about 20% on HEK293 cells, where treatment with high doses, *i.e.*, 8 μM of DOX 30% of cell cytotoxicity was observed. Treatment with IC_30_ (2 μg ml^−1^) concentration of BTE-GNP combined with IC_80_ (2 μM) of DOX resulted in about 96% cell survival, with no such cell cytotoxicity.

### 3.3 BTE-GNP induces ROS generation

**Fig. 4:**
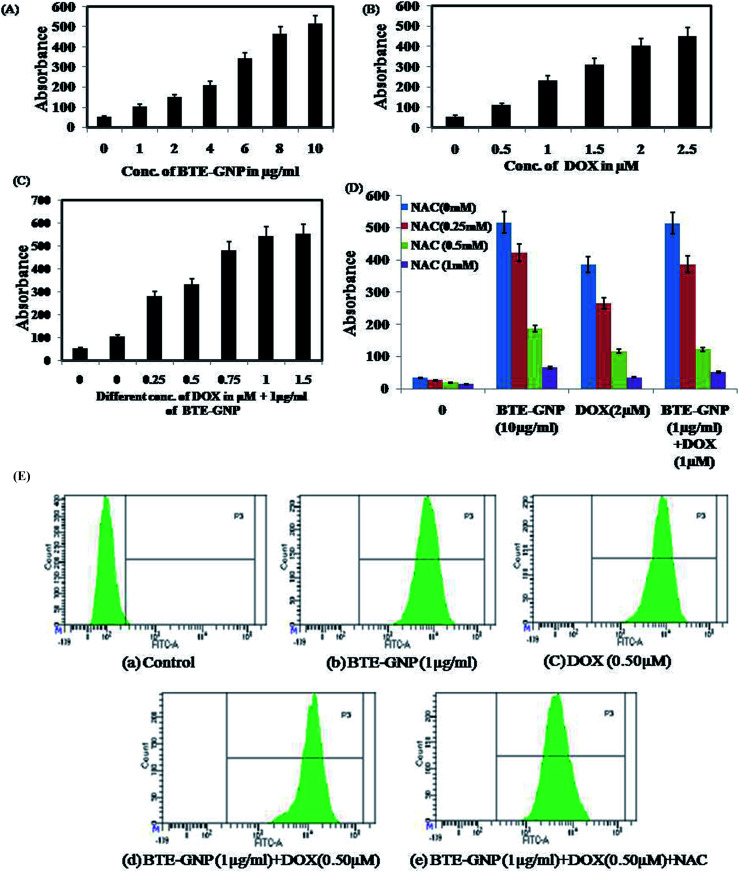
ROS level measured spectrophotometrically. HCT116 cells were exposed with the indicated doses of (a) BTE-GNP, (b) DOX, (c) BTE-GNP + DOX, and also (d) with BTE-GNP + DOX + NAC for 24 h. Here data represented are the mean ± sd of three independent experiments. (e) Level of ros production induced by BTE-GNP, DOX and BTE-GNP + DOX on HCT116 cells. HCT116 cells were treated with indicated doses of BTE-GNP, DOX, BTE-GNP + DOX and BTE-GNP + DOX + NAC for 24 h and were analyzed by flow-cytometry. Data presented here are the representation of three independent experiments.

**Fig. 5:**
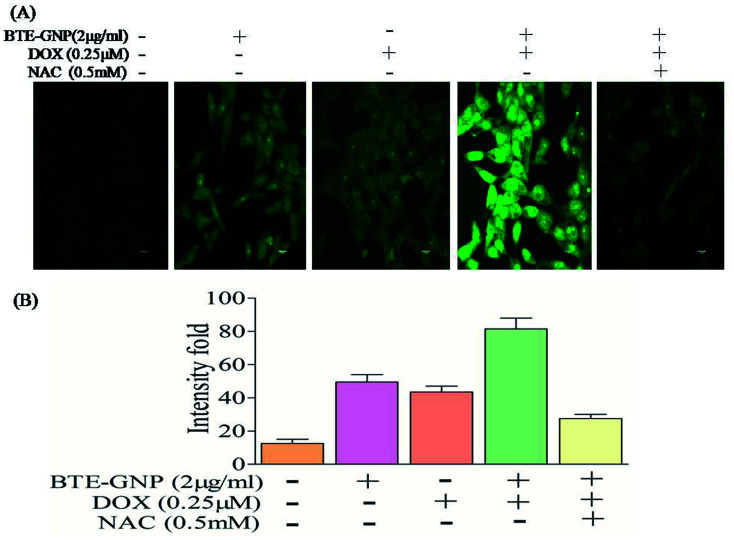
(a) Ros level observed by confocal microscopy. HCT116 cells were treated with the indicated doses of BTE-GNP, DOX, BTE-GNP + DOX and BTE-GNP + DOX + NAC for 24 h. (b) Represents the graphical parameters of the above treated values. Data presented here are the representation of three independent experiments.

Gold nanoparticle-induced ROS generation plays a vital role in its cancer cell killing.^24^ BTE-GNP, DOX and its combination induced ROS generation in HCT116 cells in a dose-dependent manner (Fig. 4A–C). ROS generation was higher with the combination of BTE-GNP and DOX. BTE-GNP + DOX-mediated ROS production was effectively antagonized by the NAC (Fig. 4D). Enhanced ROS production by BTE-GNP or its combination with DOX and inhibition by NAC was verified by flow cytometric analysis (Fig. 4E). Both the upregulated ROS generation by combined effects of BTE-GNP with DOX and also effects of NAC on the ROS level were observed by confocal microscopy (Fig. 5).

### 3.4 BTE-GNP-induced cell death is ROS dependent

**Fig. 6:**
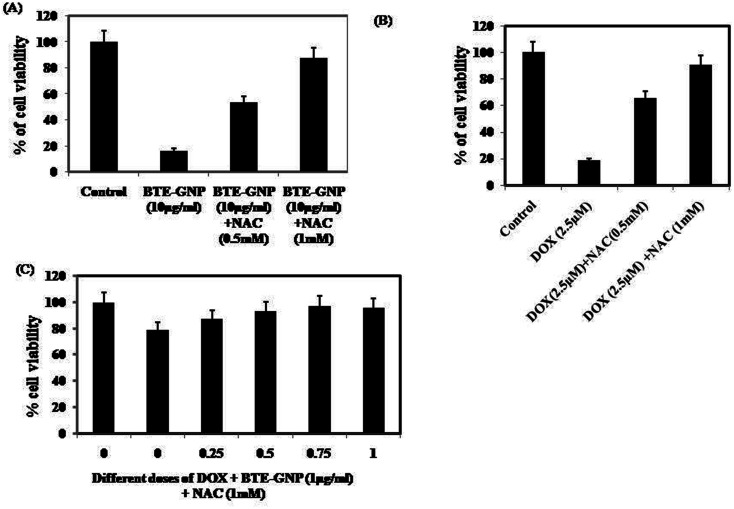
Cell survival in presence of NAC in HCT116 cells. Treated with (a) BTE-GNP (b) DOX and (c) BTE-GNP + DOX. Cytotoxicity were determined by MTT assay. Here data represented are the mean ± standard deviation of three independent experiments.

We investigated the involvement of ROS in BTE-GNP-induced cytotoxicity of HCT116 cells. As shown in Fig. 6A–C, the cytotoxicity of BTE-GNP or DOX alone and their combination was dose dependently inhibited by NAC. Therefore, combined cytotoxicity of BTE-GNP and DOX was ROS dependent.

### 3.5 BTE-GNP and BTE-GNP + DOX induces apoptotic death in HCT116 cells

**Fig. 7:**
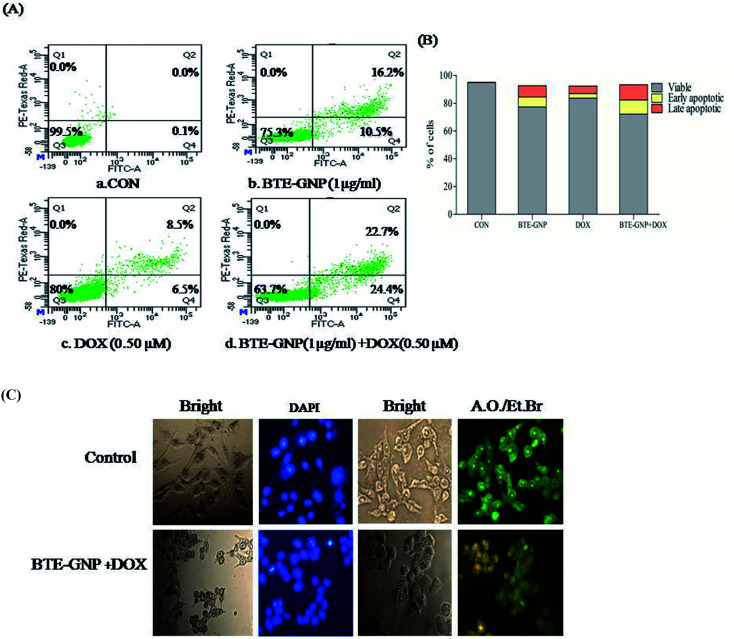
Comparative study of apoptotic death induced by BTE-GNP, DOX and BTE-GNP + DOX on HCT116 cells. (A) HCT116 cells were treated with the free DOX, BTE-GNP and combined doses BTE-GNP + DOX and were analyzed by flow-cytometry using Annexin-PI staining. (B) Represents the graphical parameters of these. Data presented here are the representation of three independent experiments. (C) Morphological changes of HCT116 induced by. HCT116 cells were treated with concentration of BTE-GNP (IC_20_: 1 μg ml^−1^) + DOX (IC_20_: 0.5 μm) and stained with dapi or AO/EtBr. The images were taken with fluorescence microscope. Images represent one of the three independent experiments.

Normally, ROS-mediated cancer cell death involves apoptosis.^25^ Therefore, involvement of apoptosis in BTE-GNP and BTE-GNP + DOX-induced cell death were verified. The percentage of apoptotic cell population was higher in BTE-GNP, DOX and BTE-GNP + DOX-treated cells than the control cells (Fig. 7A). Thus, BTE-GNP and BTE-GNP + DOX-induced cell death involved apoptosis. Fig. 7B shows the morphological change characteristics of apoptosis such as cell shrinkage, cell rounding and chromatin condensation (Fig. 7B). AO/EtBr treatment showed green fluorescence in normal cells and yellowish green colour in BTE-GNP + DOX-treated apoptotic cells.

### 3.6 BTE-GNP together with DOX alters the level of pro- and anti-apoptotic proteins in HCT116 cells

**Fig. 8:**
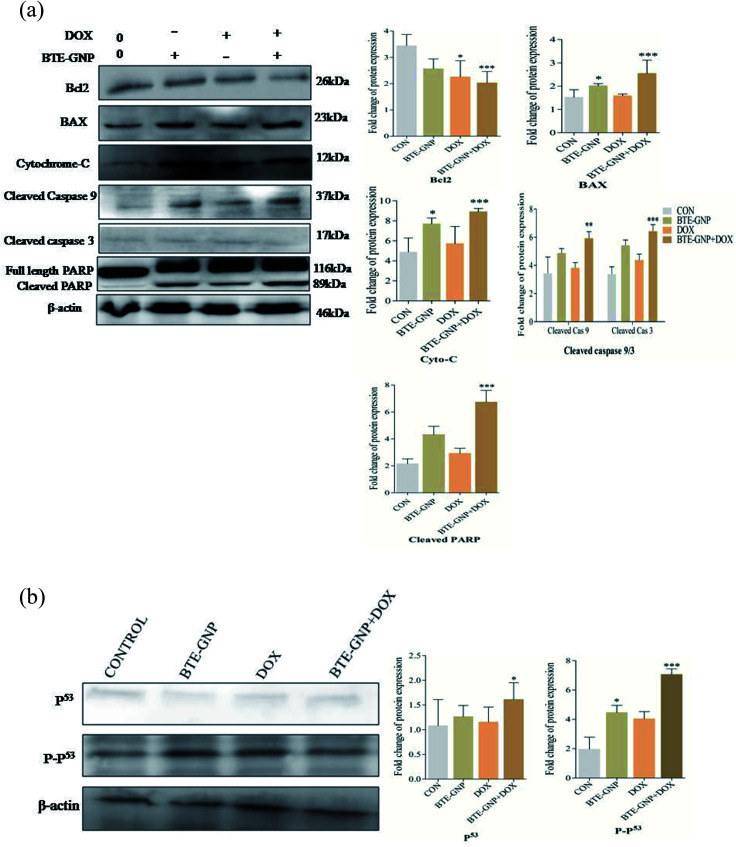
(a) Changes in the level of pro- and anti-apoptotic proteins induced by the compounds in HCT116 cells. Western blot analysis were performed of HCT116 cells treated with BTE-GNP, DOX, and BTE-GNP + DOX and some selected pro- and anti-apoptotic markers were assayed by western blot analysis as described in the ‘Materials & methods’ section. β-Actin served as a loading control levels. Right side showed densitometry graphical representation with above indicated proteins. All the experiments were done in triplicate and are reported as the mean ± sd of triplicate experiments. * Depicts the significant *p* < 0.05. (b) Expression of apoptosis inducing protein, p53 expression profiles in treated with BTE-GNP, DOX, BTE-GNP + DOX. Western blot analysis evaluated the level for using cell lysate treated with proteins as p53, P-p53 levels of the HCT116 cells as treated with BTE-GNP and DOX and BTE-GNP + DOX for 24 h. Treatment followed IC_20_ doses. All the experiments were done in triplicate and are reported as the mean ± sd of triplicate experiments. * Indicates the significant *p* < 0.05.

BTE-GNP, DOX and also BTE-GNP + DOX increased the expression of pro-apoptotic proteins such as BAX, and of cytosolic cytochrome *c*, active caspase-3/-9, cleaved PARP and anti-apoptotic protein Bcl2 compared to the untreated control, as shown in western blot analysis (Fig. 8A). Similarly, Fig. 8B indicates the higher activation level of P^53^ and also P-p53 upon treatment with BTE-GNP, DOX and also BTE-GNP + DOX. These results suggest that BTE-GNP, DOX and BTE-GNP + DOX-induced cytotoxicity occurs through enhancement of pro-apoptotic proteins and apoptotic factors.

## 4. Discussion

Proper designing of nanoparticles provide improvements on drug delivery system to treat different types of major health disorders. Here, we developed cost-effective green synthesized nanoparticles that show improved dose–response. Here, BTE-GNP synthesis using black tea extract is a very simple method and environment friendly, free from chemical hazards thus reducing the toxicity. In very recent years, combination therapy has been applied for cancer chemoprevention.^26^ Different chemosensitizers especially of natural origins are being studied to enhance the effectiveness of the existing chemotherapeutic drugs.^27^ Our study showed the bio-reduction property of ethno pharmacologically important tea leaf extract upon gold ions which had transformed into gold nanoparticles. We have used the chemotherapeutic drug doxorubicin that was sensitized by black tea-reduced GNP. Combination of DOX with BTE-GNP enhanced the cytotoxicity of HCT116 cells *via* apoptosis. Our studies have shown that DOX exhibited higher cytotoxic activity in colon cancer cells in the presence of BTE-GNP rather than monotherapy. The IC_50_ value of only black tea extract (BTE) is 950 μM. The same for BTE-GNP is 4 μg ml^−1^. Therefore, the IC_50_ value of black tea extract was drastically reduced in its gold nanoparticle (BTE-GNP) form (nearly 200 times). Combined treatment of BTE-GNP with DOX upon normal cells HEK293 provided above 95% cell survival, and no such cell cytotoxicity was found.

In this study, we had prepared GNPs using tea polyphenols as capping agents, which showed the various average sizes under acidic PBS and basic buffers and also showed uniform surface charge distribution. It has been shown that the cell viability was remarkably diminished due to combined treatment of BTE-GNP with DOX rather than the groups treated alone. The given results indicated that in the combined form BTE-GNP could induce the cytotoxicity of DOX but reducing the doses. Present data have provided the synergistic effect between BTE-GNP and DOX.

The regulation of ROS levels is a potential way to kill cancer cells without causing remarkable toxicity to normal cells.^28^ Several studies had shown that most of the anticancer agents kill cancer cells *via* excessive ROS production. BTE-GNP or DOX alone and their combination activate ROS generation that caused cell death. Dose-dependent inhibition of BTE-GNPs or DOX or BTE-GNPs + DOX induced cell death by NAC, which indicates the involvement of ROS in cell death. BTE-GNP or DOX both initiate the apoptosis of cancer cells. Researchers have shown that excessive accumulation of ROS damages mitochondria, and ultimately releases cytochrome c into the cytosol and induced apoptosis.^29^ Recently, it has been demonstrated that intracellular ROS generation plays an important role in inducing apoptosis *via* cytochrome *c*-mediated caspase 3/9 activation and PARP activation leading to cell death.^30^ BTE-GNP or DOX alone or their combination induces cytotoxicity in HCT116 cells *via* apoptosis as it was observed by morphological changes and Annexin-V binding study. In the present study, BTE-GNP and DOX upregulated the BAX, down-regulated Bcl2, released cytosolic cytochrome *c* that activated caspase-9 and caspase-3 and its downstream effector cleaved PARP. Additionally, increased intracellular ROS induces apoptosis *via* upregulation of the P-p53 protein.^31^ The P-p53 protein activated by cell stress and damage directly correlates with ROS generation, which plays an important role in the apoptotic pathway.^32^ In our study, BTE-GNP, DOX or BTE-GNP + DOX significantly upregulated P-p53, resulting in enhancement of apoptosis.

## 5. Conclusions

In conclusion, our study has suggested that BTE-GNP enhances the cytotoxic effect of DOX. Lower doses of combined treatment provided greater cytotoxicity in HCT116 cells. Therefore, BTE-GNP could be a good chemosensitizer and also a potential anticancer agent to treat the colon cancer HCT116 cells. Our study also suggests that BTE-GNP + DOX induced HCT 116 cell death *via* an ROS-dependent mitochondrial pathway of apoptosis.

## Author contributions

TD: author performed all the experiments, data analysis, written the manuscript, designed the experiments. SM: performed some revision experiments. SN: done some data analysis. KDS: designed the experiment, written the manuscript.

## Conflicts of interest

The authors do not have any conflicts to declare.

## Supplementary Material

RA-012-D1RA08374K-s001
