# Supplementary material for: Green-synthesized gold nanoparticles from black tea extract enhance the chemosensitivity of doxorubicin in HCT116 cells via a ROS-dependent pathway
Source: RSC Adv. 2022 Mar 23;12(15):8996–9007. doi: 10.1039/d1ra08374k (PMC8985146; doi:10.1039/d1ra08374k)

Electronic Supplementary Material (ESI) for RSC Advances.
This journal is © The Royal Society of Chemistry 2022

Raw data of Western Blot

Below figures represents the apoptotic proteins on HCT 116 cells obtained by Western Bolt.

1. Anti-apoptotic protein

a. (Bcl2)

2. Pro-apoptotic proteins

b. (BAX)

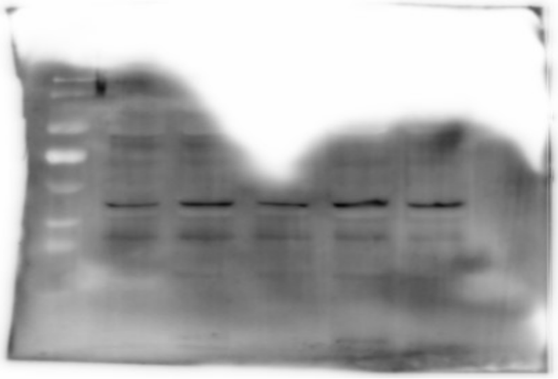

c. (Cytochrome-C)

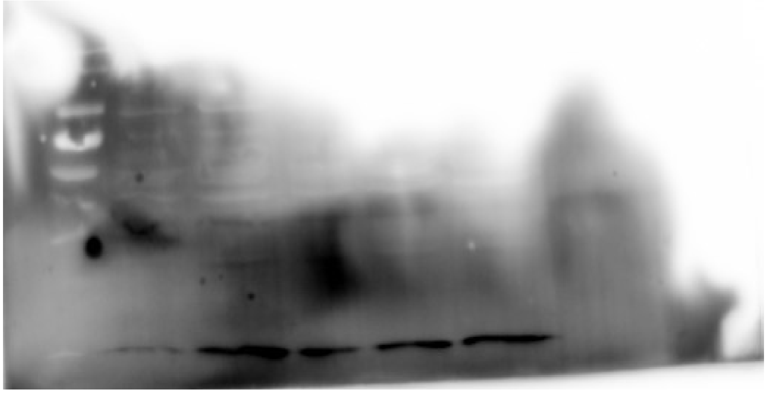

d. (Caspase-3)

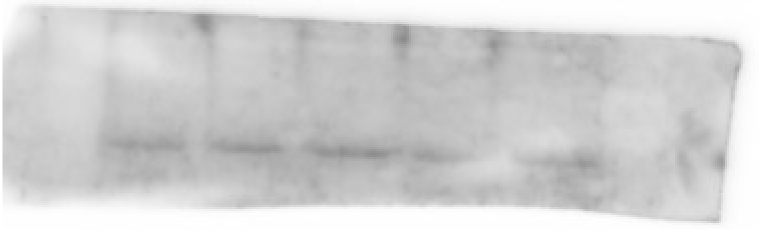

e. (Caspase-9)

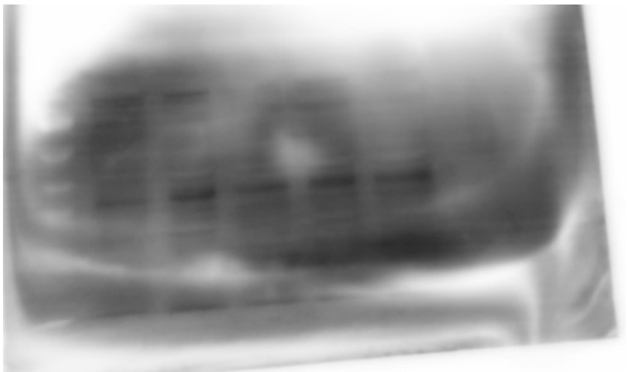

f. (PARP)

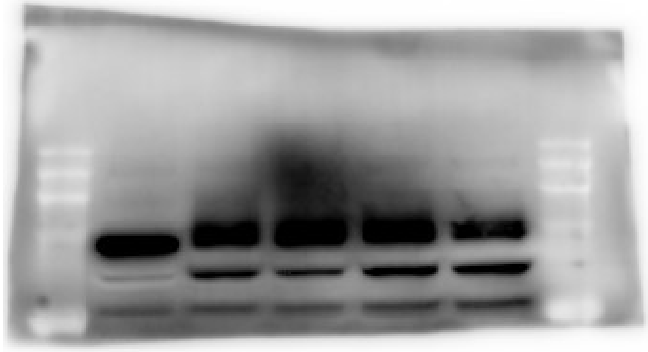

3. Cell stress induced Apoptotic (tumor suppressor protein) proteins

g. (p53)

h. (p-p53)

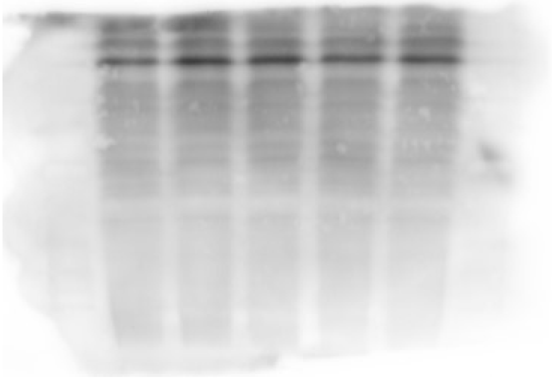

4. Normal control cellular protein as below

i. (Beta-actin 1 (figure 8A)

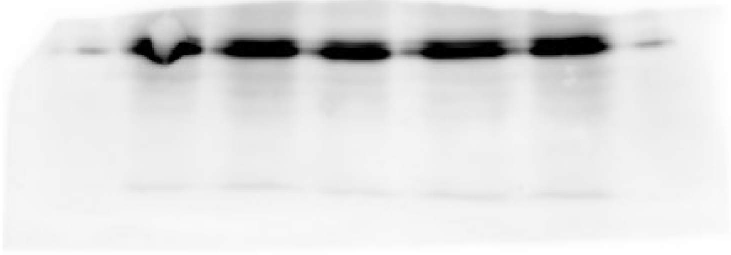

j. (Beta-actin 2 (figure 8B)

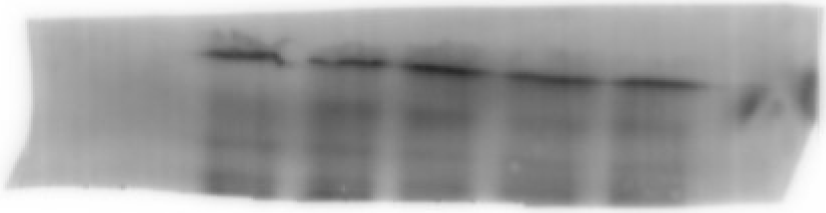

Raw data of confocal microscopy

(2) Below figures represents the ROS level on HCT 116 cells observed using Confocal microscopy.

a. (Control - HCT 116 cells)

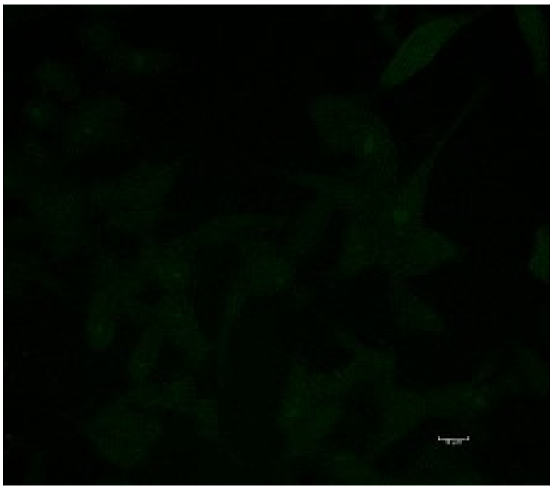

b. (BTE-GNPs treatment on HCT 116 cells)

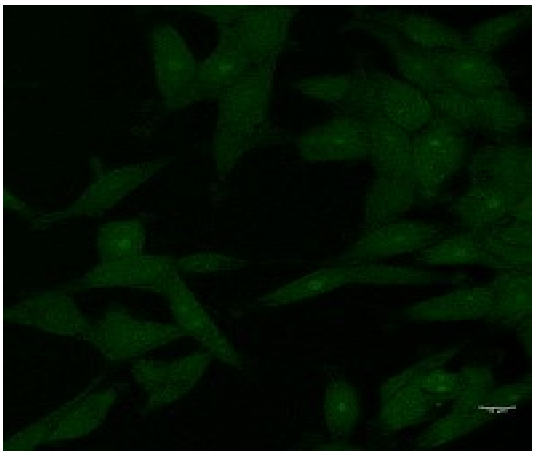

c. (DOX treatment on HCT 116 cells)

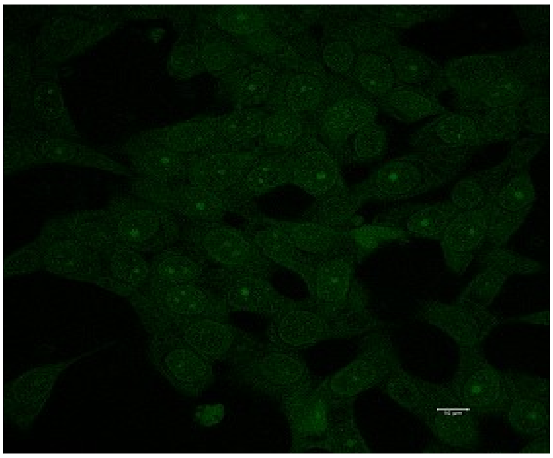

d. (Combined treatment of BTE-GNP+DOX on HCT 116 cells)

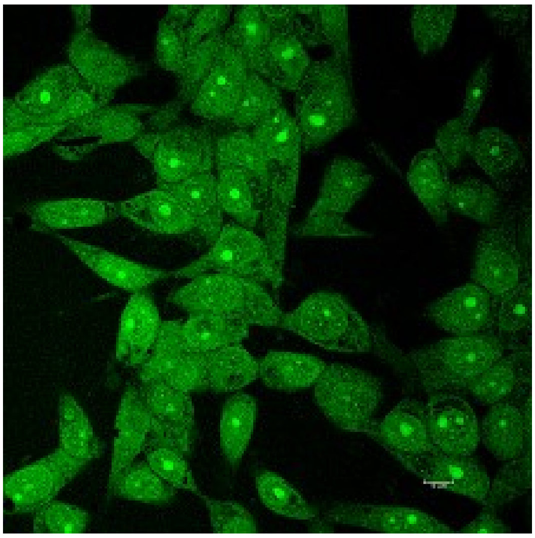

e. (Combined treatment of BTE-GNP+DOX+NAC on HCT 116 cells)

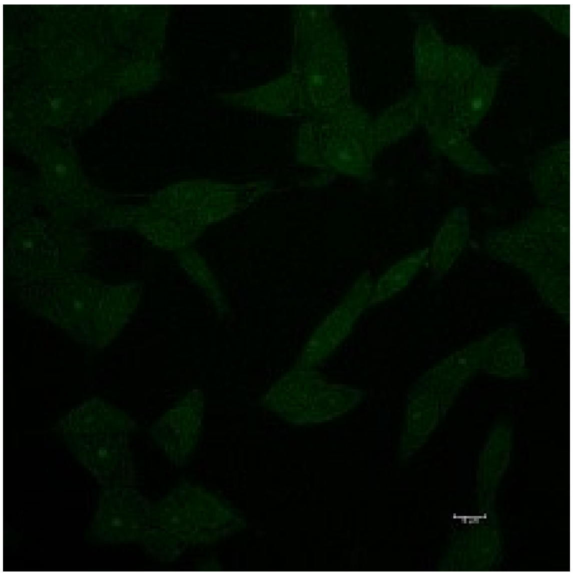

Supplement: RA-012-D1RA08374K-s001 [file RA-012-D1RA08374K-s001.pdf]
